# Different responses of soil fungal and bacterial communities to nitrogen addition in a forest grassland ecotone

**DOI:** 10.3389/fmicb.2023.1211768

**Published:** 2023-09-06

**Authors:** Daiyan Li, Meng Meng, Baihui Ren, Xinwei Ma, Long Bai, Jiahuan Li, Guohua Bai, Fengjun Yao, Chunming Tan

**Affiliations:** ^1^College of Horticulture, Shenyang Agricultural University, Shenyang, Liaoning, China; ^2^Zhangwu County Forest and Grass Development Service Center, Fuxin, Liaoning, China

**Keywords:** nitrogen addition, soil bacteria, soil fungi, microbial diversity, forest-grassland ecotone

## Abstract

**Introduction:**

Continuous nitrogen deposition increases the nitrogen content of terrestrial ecosystem and affects the geochemical cycle of soil nitrogen. Forest-grassland ecotone is the interface area of forest and grassland and is sensitive to global climate change. However, the structure composition and diversity of soil microbial communities and their relationship with soil environmental factors at increasing nitrogen deposition have not been sufficiently studied in forest-grassland ecotone.

**Methods:**

In this study, experiments were carried out with four nitrogen addition treatments (0 kgN·hm^−2^·a^−1^, 10 kgN·hm^−2^·a^−1^, 20 kgN·hm^−2^·a^−1^ and 40 kgN·hm^−2^·a^−1^) to simulate nitrogen deposition in a forest-grassland ecotone in northwest Liaoning Province, China. High-throughput sequencing and qPCR technologies were used to analyze the composition, structure, and diversity characteristics of the soil microbial communities under different levels of nitrogen addition.

**Results and discussion:**

The results showed that soil pH decreased significantly at increasing nitrogen concentrations, and the total nitrogen and ammonium nitrogen contents first increased and then decreased, which were significantly higher in the N10 treatment than in other treatments (N:0.32 ~ 0.48 g/kg; NH_4_^+^-N: 11.54 ~ 13 mg/kg). With the increase in nitrogen concentration, the net nitrogen mineralization, nitrification, and ammoniation rates decreased. The addition of nitrogen had no significant effect on the diversity and structure of the fungal community, while the diversity of the bacterial community decreased significantly at increasing nitrogen concentrations. Ascomycetes and Actinomycetes were the dominant fungal and bacterial phyla, respectively. The relative abundance of Ascomycetes was negatively correlated with total nitrogen content, while that of Actinomycetes was positively correlated with soil pH. The fungal community diversity was significantly negatively correlated with nitrate nitrogen, while the diversity of the bacterial community was significantly positively correlated with soil pH. No significant differences in the abundance of functional genes related to soil nitrogen transformations under the different treatments were observed. Overall, the distribution pattern and driving factors were different in soil microbial communities in a forest-grassland ecotone in northwest Liaoning. Our study enriches research content related to factors that affect the forest-grassland ecotone.

## 1. Introduction

Since the industrial revolution, atmospheric nitrogen (N) deposition has increased globally due to human activities, such as industrial and agricultural production, fertilizer use, animal husbandry development, and land use change. Atmospheric N content has increased by three- to fivefold in the twentieth century, and the growth rate of global N deposition is expected to reach 1–2 times that of the 2000s by the 2050s (Davidson, 2009). The level of N deposition in China is far higher than the global average. Increased atmospheric N deposition affects both human health and the environment (Eisenlord et al., 2013). Atmospheric N deposition interferes with the N cycle in terrestrial ecosystems by fixating soil carbon (Bai et al., 2010). Excess N deposition leads to soil acidification and changes in plant composition and affects the soil N cycle, thus affecting ecosystem characteristics (BassiriRad, 2015). Therefore, N deposition is a global environmental problem.

Soil microorganisms are the most active components in terrestrial ecosystems. They not only undertake important tasks, such as decomposing plant and animal remains, but also promote the material cycle and energy flow of the ecosystem (Muhammad et al., 2023). The soil microbial structure and diversity are important indicators of soil quality (Morris and Blackwood, 2015). Among the soil microorganisms, bacteria and fungi play an important role in material transformation. Addition of N further changes the structure and diversity of the soil microbial community by changing the original physical and chemical properties of the soil (Hao et al., 2018). Studies have shown that the structure of the soil microbial communities in temperate steppes was considerably changed when supplemental levels of N were greater than 120 kg N·hm^−2^·a^−1^ (Zeng et al., 2016). Long-term N addition has been shown to improve the soil microbial diversity in Swedish grasslands (Freitag et al., 2005). However, Ling et al. (2017) arrived at the opposite conclusion in their study on the effects of long-term N addition in a temperate steppe in Inner Mongolia, China. Soil microbial richness and diversity indices were negatively correlated with the level of N addition. Similarly, Wang H. et al. (2018) found that addition of N markedly reduced the soil microbial diversity in tropical forest soils in southern China. Yang et al. found that short-term application of N had no substantial effect on the soil microbial community structure (Yang et al., 2015), meanwhile, a previous study showed that short-term nitrogen addition significantly altered soil bacterial community in an alpine steppe on the Tibetan Plateau (Huang et al., 2022). Therefore, the results of nitrogen addition on microorganisms in different ecosystems are inconsistent, so further studies are needed to clarify the effects on microorganisms in our forest grassland ecotone ecosystems.

Different soil microbial groups respond differently to the addition of N. Studies have shown that N addition can affect the abundance of the dominant soil bacterial flora and change the structure of the soil bacterial community (Hongli et al., 2022). Other studies have shown that short-term application of N has no substantial effect on soil bacterial communities (Zhao, 2022); however, treatment with high levels of N lead to considerable changes in the relative abundance of sensitive bacterial communities, resulting in an increase in the abundance of eutrophic bacteria and a decrease in the abundance of hypotrophic groups. Ammonium N is an important source of N for microorganisms, and its content affects the growth of microorganisms sensitive to it. Zhou et al. showed that the increase in the abundance of Acidomycetes and α-Proteobacteria was related to the increase in ammonium N content. Fungal communities may be more sensitive to N addition than are bacterial communities. Many studies have shown that N addition markedly reduces the richness and diversity of ectomycorrhizal fungi and alters their community composition. High-throughput sequencing has shown that Ascomycota and Zygomycota were the dominant phyla in soil fungi. Input of N affected the relative abundance of fungal community groups. For example, elevated soil N content (due to N input) increases the relative abundance of Ascomycota. Some studies have shown that addition of N leads to soil acidification, which is an important factor that causes changes in the structure of the soil microbial community. However, other studies have shown that the increase in available N content in the soil caused by N deposition leads to changes in the soil microbial community structure rather than soil acidification. Yuan Fang et al. showed that the soil water content and total soil carbon and N (TN) contents affect the composition of the soil microbial community in a meadow steppe. Therefore, the influence of N deposition on soil microbial communities and its driving factors are highly uncertain and require further study.

High N deposition causes changes in the soil N conversion process (Yang et al., 2016), increases the available N content in the soil (Zhang and Han, 2012), and changes the number and composition of N-cycling microorganisms, thus affecting the ecosystem. The main processes involved in soil N conversion include biological N fixation, ammonification, nitrification, and denitrification. A series of transformations after N deposition in the soil cannot be achieved without the participation of related microorganisms. Since N fixation is an energy-consuming process, it is generally believed that the number of N-fixing microorganisms is positively correlated with the content of the organic matter in the soil. The addition of N fertilizers increased the soil organic carbon content and the abundance of Azotobacter, thus promoting the N fixation function of soil microorganisms. Recent studies have shown that the addition of N considerably affects the community structure and abundance of Azotobacter, nitrifying, and denitrifying bacteria (Jorquera et al., 2014). The abundance of Azotobacter and the N-fixing function of soil microorganisms were improved by adding an appropriate amount of N (Orr et al., 2012). However, the addition of high concentrations of inorganic N inhibited the growth of Azotobacter, thus inhibiting the growth of N-fixing microorganisms. Ning et al. (2015) found that N-related functional genes showed different sensitivities to the level of N addition, and the AOB-*amoB* gene of ammonia-oxidizing bacteria was more sensitive to N addition than the AOA-*amoA* gene of ammonia-oxidizing archaea. AOB abundance increased with an increase in N content, but AOA abundance did not change considerably (Shen et al., 2011).

China extends over a vast territory and has rich natural grassland resources, with nearly 400 million hectares of grassland area, accounting for 41% of China’s total land area (Zhang et al., 2016). It is the world’s second largest grassland country, and the forest-grassland ecotone is located in the transition zone between forest and grassland vegetation types; however, the coexistence of forest and grassland vegetation is characterized by vegetation type. The forest-grassland ecotone in northwest Liaoning is part of the forest-grassland ecotone in northeast China. The forest-grassland ecotone is a typical biodiversity-rich area that is sensitive to climate change, but the overall understanding of the soil microbial communities is relatively limited. In the context of increasing N deposition, it remains unclear whether the composition and diversity of the soil microbial communities in forest-grassland ecotone have unique response characteristics.

To understand the characteristics and factors that influence the soil microbial communities in the forest steppe ecotone of northwest Liaoning under different N concentrations, we used high-throughput sequencing and quantitative (q) PCR technologies to answer three questions: (1) Do different N concentrations affect the soil physicochemical properties? (2) Do different N concentrations affect the composition of the soil bacterial and fungal communities and the abundance of N-transforming functional microorganisms? (3) What are the potential mechanisms by which environmental factors affect the soil microbial communities in the presence of increased N deposition? Based on previous studies, we hypothesized that: (a) Treatments with different N concentrations would affect the soil physicochemical properties; (b) Notable differences might be observed in the composition of the bacterial and fungal communities and the abundance of functional microorganisms involved in N transformation after treatment with different N concentrations; and (c) Changes in soil TN, pH, and other environmental factors would lead to changes in the soil microbial communities.

## 2. Materials and methods

### 2.1. Description of study area

The study area is located in the Zhangwu village of northern Liaoning Province, China (42° 08′–42° 50´ N, 121° 53′–122° 58′ E). This region has a semiarid climate with an average annual precipitation of 450–500 mm, annual pH of 5.98–6.35 and average annual temperature of 6.2–7.2°C. Sixty percent of rainfall occurs between June and August.

The experimental site was located in the transition zone from the western grassland to the eastern forest, and the vegetation is a community of trees, shrubs, and herbs. The predominant tree species are *Crataegus pinnatifida*, *Ulmus pumila*, *Populus simonii*, and *Salix matsudana*. Shrubs, including *Ulmus davidiana* Planch, *Armeniaca sibirica*, *Rhamnus parvifolia* Bunge, and *Lespedeza daurica*, are also present. Herbs include *Cleistogenes squarrosa*, *Potentilla tanacetifolia*, *Agropyron cristatum*, and *Artemisia frigida*.

### 2.2. Experimental design and sample collection

The experiment was conducted using a one-factor randomized design comprising four treatments. N was applied at rates of 0 (N0), 10 (N10), 20 (N20), and 40 kg N·hm^−2^·a^−1^ (N40), with five replicates per treatment. Each plot had an area of 2 m^2^ × 2 m^2^, with a 2 m-wide buffer strip separating each plot. The rates of N addition were determined based on global N-deposition levels. N was added in the form of urea (CO(NH_2_)_2_) three times in May, June, and August, 2016. The amount of fertilizer applied each time was 1/3 of the total amount applied throughout the year (Weigh on a ten-thousandth balance). The fertilizer was dissolved in 6 L of tap water and sprayed evenly in the quadrat using a watering can. For the N0 treatment, the same amount of water was used.

After 4 years of N addition, soil samples were collected in September 2020 at depths of 0–20 cm. Five soil cores from randomly selected locations in each plot were mixed to form a composite sample (Random sampling method). Small stones, roots, and litter were removed from the composite samples, and the soil was divided into two subsamples. One subsample was air-dried and sieved through a 0.25-mm mesh for physicochemical analysis. The other subsample was placed in bags with ice and immediately transferred to a super-cold refrigerator (−80°C) for DNA extraction.

### 2.3. Soil physicochemical analysis

Soil total nitrogen and total phosphorus contents were measured using SmartChem140 intermittent analyzer (EL Ш, Elementar, Germany) with indophenol blue colorimetric method and Mo-Sb colorimetric method, respectively. Soil organic carbon content was determined by the K_2_Cr_2_O_7_ oxidation method. Soil pH was determined using the glass electrode method, and conductivity was determined using a conductivity meter. The other portion of the sample soils was stored frozen at −80°C for high-throughput sequencing and determination of ammonium nitrogen and nitrate nitrogen, which were analyzed based on a continuous-flow ion auto-analyzer (SAN++, Skakar, Breda, Holland) after extracting with 2 M KCl. Alkali-hydrolyzed nitrogen was determined by diffusion dish culture method.

### 2.4. Soil nitrogen mineralization rate

Soil N mineralization was determined using an indoor culture method. Fifteen grams of the screened air-dried soil sample was weighed and placed in a 250 mL plastic wide-mouth bottle. The water content of the soil was adjusted to 20% (mass water content) with deionized water. The bottle was wrapped with polyethylene film, and two holes were tied into the film, and the soil was pre-cultured in an incubator at 25°C for 7 days. After pre-culture, the samples were divided into three groups by adding ammonium sulfate, sodium nitrate solution, and equal weight of deionized water (control group [CK]) to 50 mg N·kg^−1^ soil (dried weight). Soil moisture in all three groups was adjusted to 40% of the soil water-holding capacity. All culture bottles were re-sealed and incubated in the dark at 25°C for 14 days. The change in soil moisture was determined by weighing, and deionized water was used to replenish moisture. On d 0 and 14 of culture, 75 mL of 2 mol·L^−1^ KCL solution was added, and the samples were incubated in a constant-temperature oscillator at 25°C for 1 h. The filtrate was treated using a continuous flow analyzer (Auto Analyzer 3; SEAL Analytical, UK) to determine the nitrate and ammonium N concentrations in the soil.

### 2.5. DNA extraction, Illumina MiSeq high-throughput sequencing, and sequence processing

The Power Soil DNA Isolation Kit (MoBio, USA) was selected to extract genomic DNA from the samples, after which the purity and concentration of DNA were tested using agarose gel electrophoresis and Nanodrop; The diluted genomic DNA was used as template for PCR using specific primers with Barcode and efficient high fidelity enzymes according to the selection of sequencing regions. We used the 336F:5´-GTACTCCTACGGGAGGCAGCA-3´ and 806R:5´-GGACTACHVGGGTWTCTAAT-3′ for bacterial amplification primers, and the fungal amplification primer was ITS4 (5´-TCCTCCGCTTA TTGATA TGC-3′)/gITS7F (5´-GTGARTCA TCGA RTCTTTG-3′); Library construction was performed using TruSeq® DNA PCR-Free Sample Preparation Kit library construction kit. The constructed libraries were quantified by Qubit and Qpcr. After the libraries were qualified, the v2 sequencing kit (2×250 bp) and Miseq sequencer were used for on-board sequencing.

The obtained raw sequence data were analyzed using the Quantitative Insights into Microbial Ecology (QIIME v.1.8.0) pipeline.^1^ Sequences were quality-filtered, denoised, merged, and chimeras were removed using the DADA2 plugin before clustering. Non-chimeric sequences were then 97% reclustered using Vsearch (v2.13.4) to generate representative operational taxonomic unit (OTU) sequences and OTU tables. The PyNAST method was used for sequence alignment. Taxonomy was assigned to amplicon sequence variants (ASVs) using the classify-sklearn Naïve Bayes taxonomy classifier in the feature-classifier plugin against the Greengenes 99% OTUs reference sequences (13_8 release). The alpha-diversity metrics (Chao1 [Chao, 1984], observed species, Shannon [Shannon, 1948a], Simpson [Simpson, 1949], Pielou’s evenness [Pielou, 1969], and Good’s coverage [Good, 1953]), and beta diversity metrics (Bray–Curtis dissimilarity) were estimated using the diversity plugin, with samples rarefied to sequences.

The DNA sequences in this study have been deposited in the National Center for Biotechnology Information (NCBI) Sequence Read Archive (SRA) database under accession number PRJNA949941.

### 2.6. Fluorescence quantitative PCR technique

Quantitative fluorescence PCR was used to determine the abundance of soil microorganisms. The reaction system was as follows: soil N-fixation, AOB, AOA, and denitrifying bacteria were used as the quantitative amplification primers (Table 1). The total volume of the amplification reaction system was 20 μL, and included 10 μL 2 × GoTaq® qPCR Master Mix, 10 μmol/L upstream and downstream primers (0.5 μL each), 2 μL DNA template (1–10 ng), and 7 μL sterilized ultra-pure water. The enhanced 96-PCR plate was amplified on a quantitative fluorescence PCR instrument, with three replicates per sample. The amplification reaction conditions were as follows: predenaturation at 95°C for 30 s, denaturation at 95°C for 5 s, annealing at 60°C for 40 s, extension at 72°C for 30 s, and 40 cycles. The amplification efficiency can be seen from Table 2. qPCR algorithm: Absolute quantification.

**Table 1:** Primers for PCR amplification.

Microbial types	Primer name	Primer sequence	Fragment length
*nifH*	Upstream	AAAGGYGGWATCGGYAARTCCACCAC	432
	Downstream	TTGTTSGCSGCRTACATSGCCATCAT	
*amoA*-AOB	Upstream	GGGGTTTCTACTGGTGGT	491
	Downstream	CCCCTCKGSAAAGCCTTCTTC	
*amoA*-AOA	Upstream	STAATGGTCTGGCTTAGACG	635
	Downstream	CACCGTTTACTGCCAGGACT	
*nirK*	Upstream	GGMATGGTKCCSTGGCA	514
	Downstream	GCCTCGATCAGRTTRTGG	

**Table 2:** Primers amplification efficiency:

gene	Slope	Y-Inter	R2	Eff%
*nifH*	−3.2782	39.209	0.9989	101.86
AOA	−3.3755	39.228	0.9984	97.81
AOB	−3.7144	42.123	0.9983	85.86
*nirK*	−3.4435	42.184	0.9982	95.17

The formulas as follows: copies = $10^{\frac{\mathrm{Ct}-b}{k}}$

### 2.7. Calculations and statistical analyses

The following procedure was used to analyze the physical and chemical properties of the soil: first, Microsoft Excel 2019 was used to sort, count, and map the measurement results for each index. SPSS 22.0 (IBM) was used for one-way ANOVA, and the data were expressed as the mean ± standard deviation (SD). The least significant difference (LSD) method was used for multiple comparisons between different treatments (*p* < 0.05).

The formulas for calculating the soil N conversion rate were as follows:


$$\mathrm{NMR}\;\left(\mathrm{mg}\cdot {\mathrm{kg}}^{-1}\cdot d^{-1}\right)=\left(\mathrm{Mt}+\mathrm{Nt}\right)-\frac{M0+N0}{t}$$

$$\mathrm{NNR}\;\left(\mathrm{mg}\cdot {\mathrm{kg}}^{-1}\cdot d^{-1}\right)=\left(N\;t-N0\right)/t$$

$$\mathrm{NAR}\;\left(\mathrm{mg}\cdot {\mathrm{kg}}^{-1}\cdot d^{-1}\right)=\left(\mathrm{Mt}-M0\right)/t$$

where NMR, NNR, and NAR are the net mineralization, nitrification, and ammoniation rates, respectively; N_0_ and N_t_ are the nitrate N concentrations at the beginning and t days after culture; and M0 and Mt. are the ammonium N concentrations at the beginning and t days after culture.

### 2.8. Statistical analyses

One-way analysis of variance (ANOVA) was performed using the software SPSS22.0 (IBM Co, Armonk, NY, USA) to determine the differences of environmental factors among different N treatments. Pearson correlation analysis was performed to explore the correlation between environmental factors and soil microbial diversity. The significance between different treatments was confirmed using Tukey’s HSD test at *p* < 0.05.

The “ggplot2” package in R was used to draw box line plots and to visualize the compositional distribution of each sample at the systematic taxonomic level to determine differences in microbial community composition and alpha diversity among different nitrogen addition levels. The structural composition of the microbial communities of the different nitrogen addition levels was visualized using principal coordinate analysis (PCoA) using the “ape” package in R language, which is based on the Bray-Curtis heterogeneity matrix. To examine the effect of nitrogen addition levels on microbial β-diversity, PERMANOVA (Permutational multivariate analysis of variance) analysis was used. The effects of environmental factors on microbial community structure were analyzed by constrained ordinal redundancy analysis (RDA) in CANOCO 5.0 software (Microcomputer Power, Ithaca, NY, USA). The effect of each variable was assessed using an RDA-based Monte Carlo test (999 permutations).

## 3. Results

### 3.1. Effects of N addition on soil physicochemical properties

Table 3 shows the addition of N significantly affected TN and NH_4_^+^-N contents and the soil pH. The TN content in the N10 treatment was significantly higher than those in the other treatments. The highest pH was recorded in the CK treatment and the lowest in the N40 treatment. However, all values were in the 6–7 range. The NH_4_^+^-N content reached a maximum in the N10 treatment and a minimum in the N40 treatment.

**Table 3:** Soil physical and chemical properties under different nitrogen addition treatments.

Soil chemical properties	Treatments			
	CK	N10	N20	N40
TN(g∙kg^−1^)	0.12 ± 0.02^c^	0.4 ± 0.08^a^	0.12 ± 0.01^c^	0.25 ± 0.08^b^
TP(g∙kg^−1^)	0.54 ± 0.11	1.19 ± 0.54	0.97 ± 0.42	0.55 ± 0.25
AP(mg∙kg^−1^)	5 ± 2.36	5.35 ± 4.57	5.18 ± 0.93	4.23 ± 0.83
SOC(g∙kg^−1^)	5.22 ± 0.86	5.79 ± 0.7	5.4 ± 1.61	5.16 ± 0.68
pH	6.69 ± 0.06^a^	6.68 ± 0.04^a^	6.46 ± 0.15^ab^	6.21 ± 0.25^b^
EC(S∙m^−1^)	16.86 ± 4.5	18.74 ± 2.75	14.51 ± 0.66	13.87 ± 2.56
NO_3_^+^-N(mg∙kg^−1^)	0.46 ± 0.02	0.53 ± 0.4	0.59 ± 0.16	0.58 ± 0.11
NH_4_^+^-N(mg∙kg^−1^)	5.11 ± 4.5^b^	12.27 ± 0.73^a^	5.81 ± 2.32^b^	2.65 ± 0.49^b^
AN(mg∙kg^−1^)	26.25 ± 3.5	33.25 ± 6.31	32.67 ± 4.4	30.33 ± 1.01

TN represents total nitrogen; TP represents total phosphorus; AP represents available phosphorus; SOC represents soil organic carbon; EC represents electrical conductivity; NH_4_^+^-N represents ammonium nitrogen; NO_3_^−^-N represents nitrate nitrogen; AN represents available nitrogen. Different letters in the same column indicate significant difference at the 0.0:5 level. The same as below.

**Figure 1:**
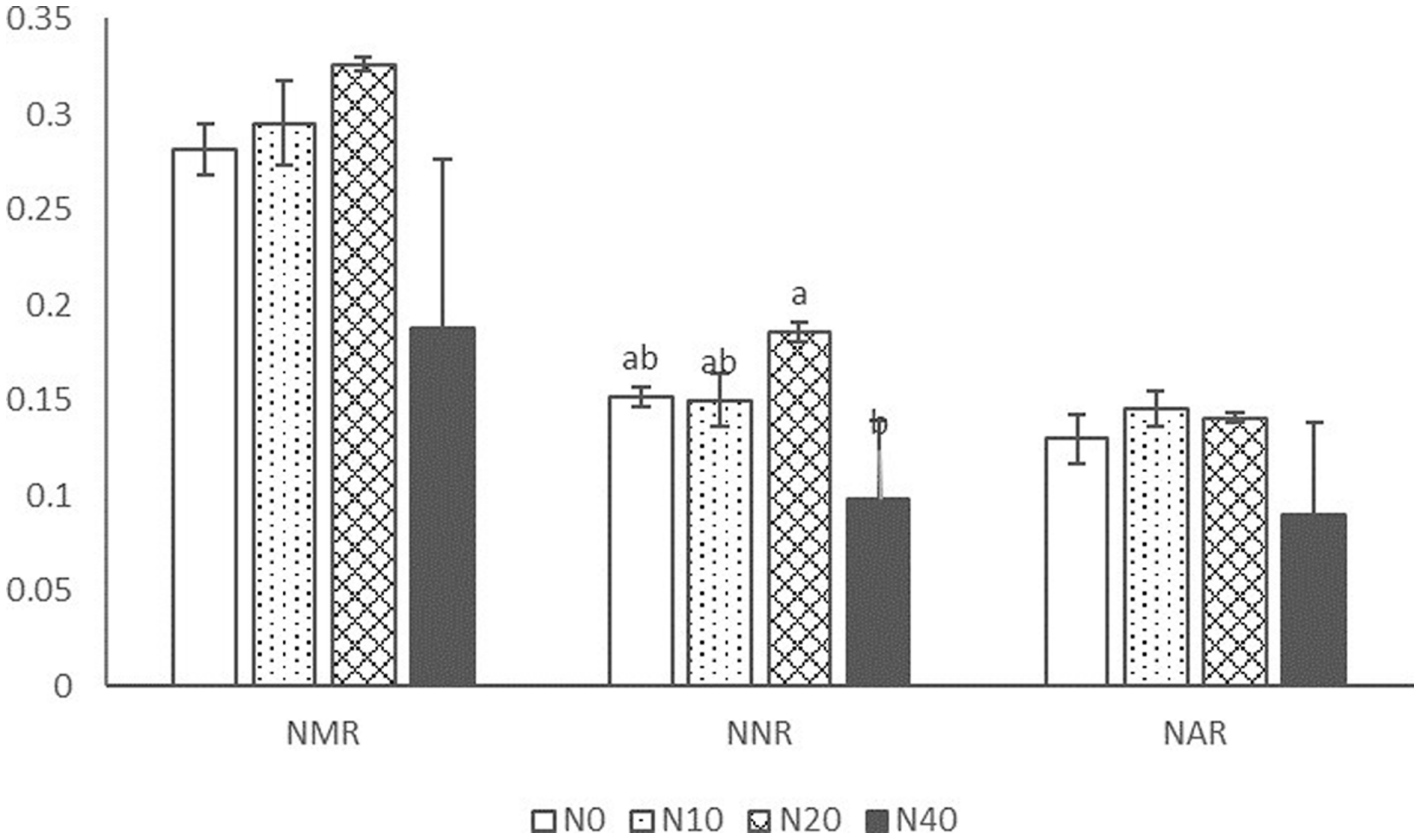
NMR, NNR, and NAR, net nitrification, ammonification, and mineralization rates; N0, N10, N20, and N40, application of 0, 10, 20, and 40 kg Nitrogen (N)·hm^−2^·a^−1^.

As shown in Figure 1, the addition of N had a significant effect only on NNR but not on other rates. NAR, NNR, and NMR first increased and then decreased with increasing N concentrations; the highest values of NMR and NNR were observed in the N20 treatment, while NAR values were the highest in the N10 and N20 treatments.

### 3.2. Diversity and composition of microbial communities

#### 3.2.1. Relative abundance of dominant phylum

**Figure 2:**
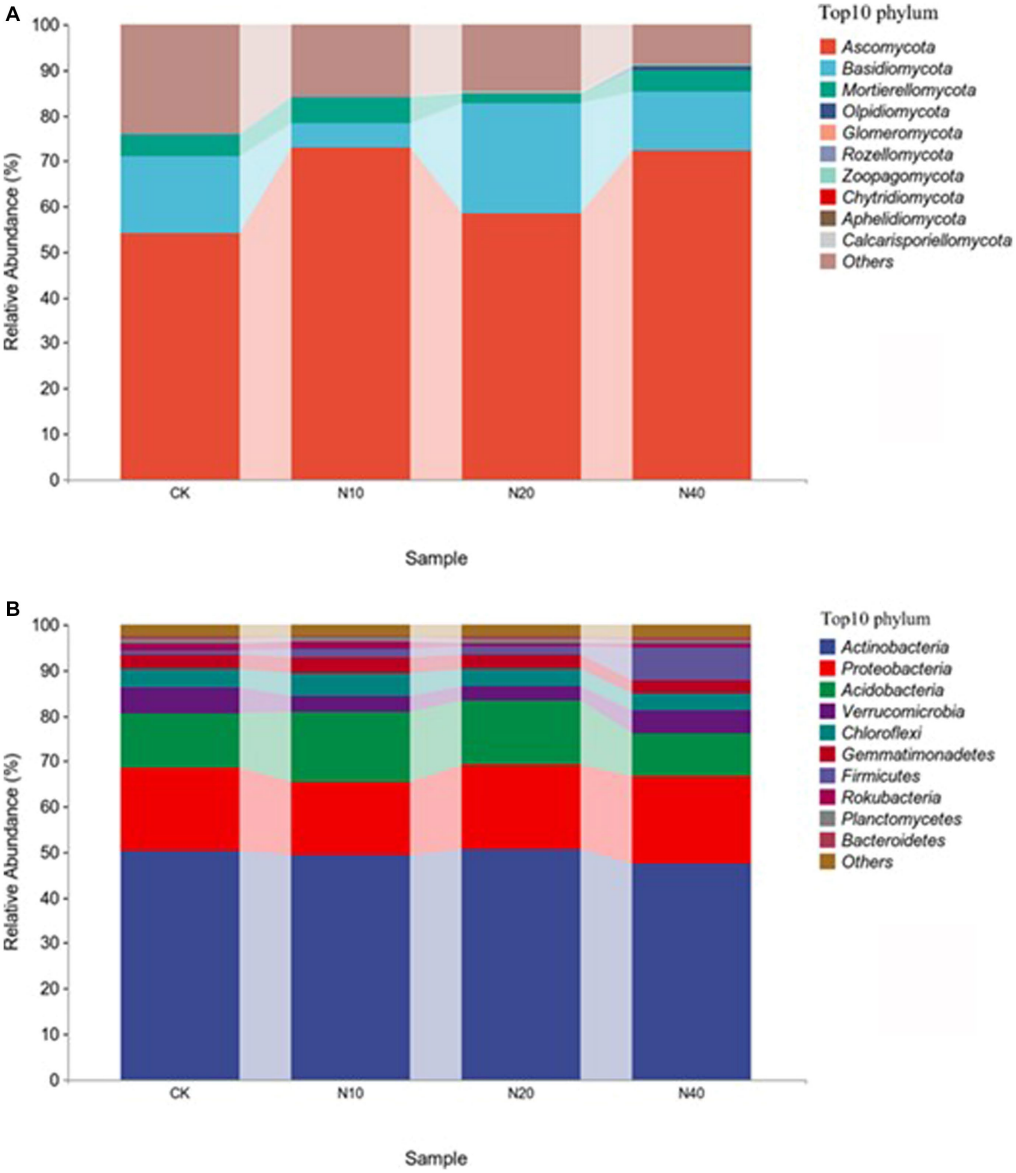
Relative abundance of **(A)** fungal and **(B)** bacterial communities at the phylum level.

Figure 2 shows the composition of bacterial and fungal communities at the phylum classification level under different N concentrations. Ascomycota, Basidiomycota, and Mortierellomycota had high relative abundances, with average relative abundance ratios of 64.45, 14.79, and 4.4%, respectively. Ascomycetes was the most dominant phylum common to the soil fungal communities, and the relative abundance ratio did not differ significantly under different N treatments. *Actinomyces*, Proteobacteria, and Acidobacteria were the most abundant bacteria, with average relative abundance ratios of 49.35, 18.06, and 12.68%, respectively. Actinomycetes was the most dominant phylum in the soil bacterial communities, and the relative abundance ratio did not differ significantly under different N treatments. The relative abundance of Acidobacteria was significantly different after treatment with different N concentrations.

**Figure 3:**
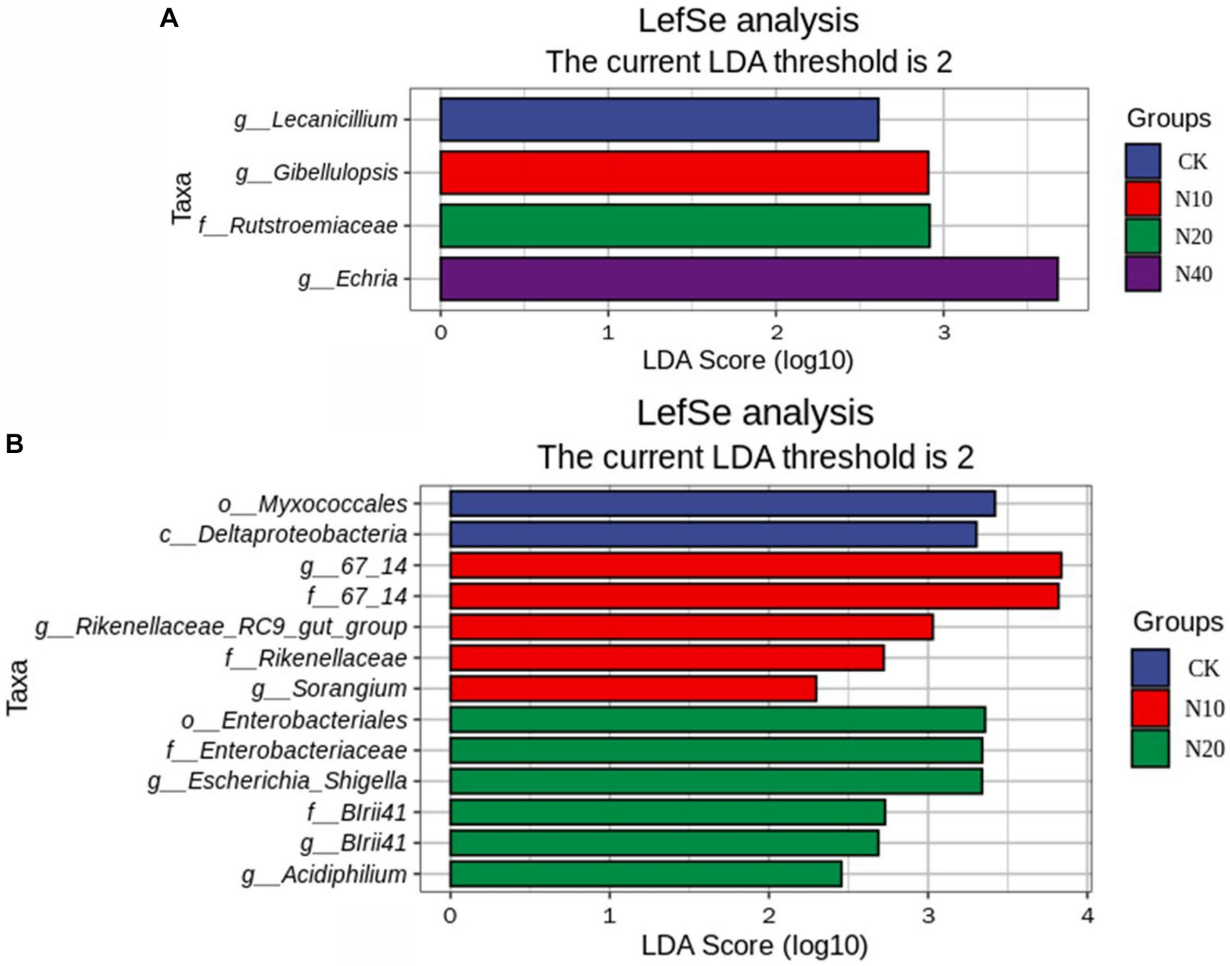
Soil fungal **(A)** and bacterial **(B)** community with linear discriminant analysis (LDA) scores.

Figure 3 shows the N addition significantly increased 2 taxa of the soil bacterial community and increased 3 taxa of the soil fungi community. The significant changes in soil bacterial taxa caused by N addition were mainly related to the *Myxococcales* and *Enterobateriales*. The significant changes in soil fungi taxa caused by N addition were mainly related to the *Lecanicillium, Gibellulopsis, Echria* and *Rutstroemiaceae.*

#### 3.2.2. α-Diversity

**Figure 4:**
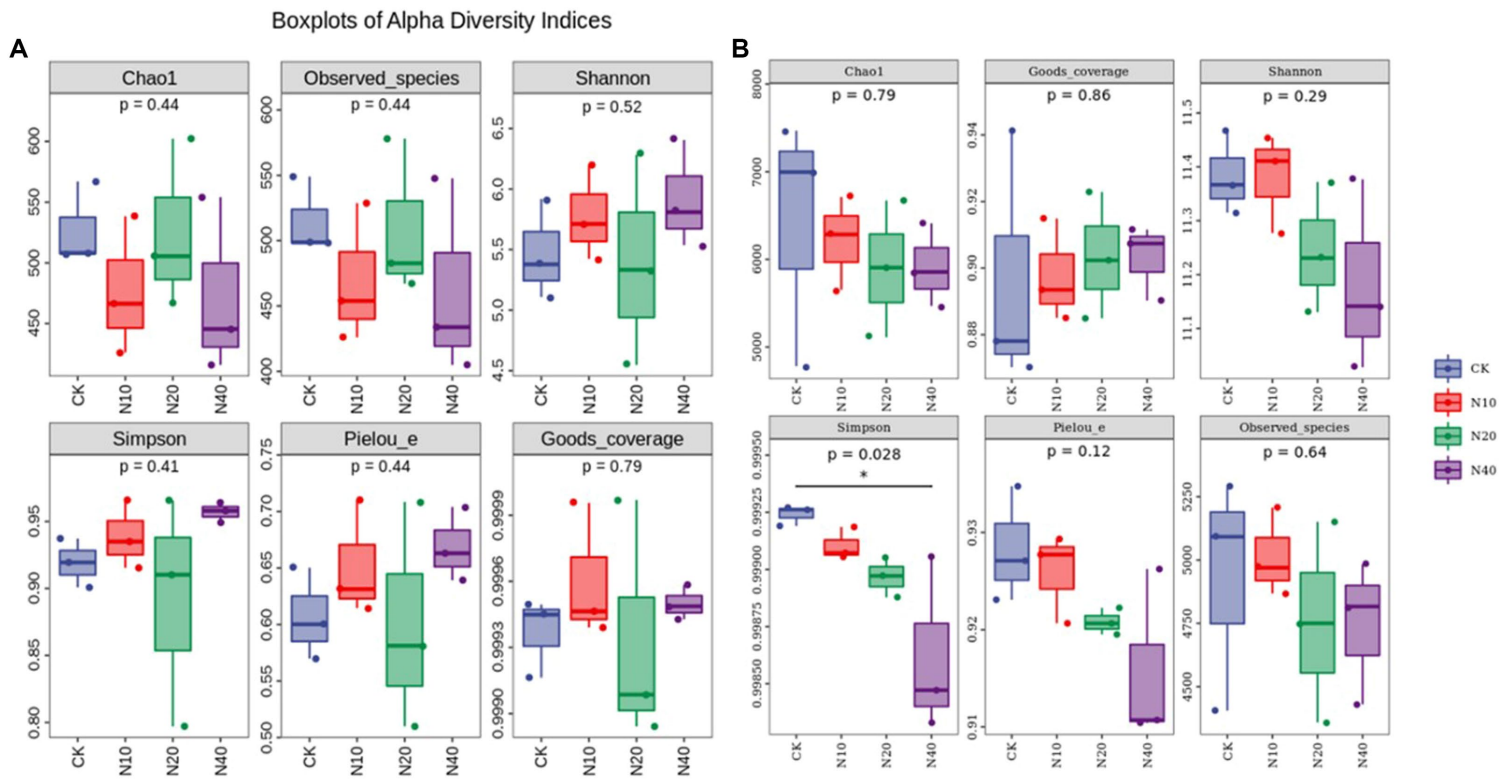
Boxplots of alpha diversity indices of **(A)** fungal and **(B)** bacterial communities.

Figure 4 shows the alpha diversity indices of fungal and bacterial communities. The Chao1 indices of the soil fungal communities under different N additions ranked as follows: N40 < N10 < N20 < CK. The uniformity of the soil fungal community (Pielou’s index) ranked as follows: N20 < CK < N10 < N40. The soil fungal community diversity (Shannon’s index) ranking order was as follows: N20 < CK < N10 < N40. The soil fungal diversity (alpha diversity) at the four N concentrations showed that, although the fungal community diversity varied with different N treatments, there were no significant differences in the Chao1, observed species, Shannon (1948a,b), and Simpson (1949) indices (*p* > 0.05).

The Chao1 indices of the soil bacterial communities at different N concentrations were in the following order: N40 < N20 < N10 < and CK. The soil bacterial community uniformity (Pielou’s index) ranked in the following order: N40 < N20 < N10 < and CK. The ranking order of the soil bacterial community diversity (Shannon index) was as follows: N40 < N20 < N10 < CK. The Simpson index was significantly different among treatments (*p* < 0.05), while no significant differences were noted in the other diversity indices.

#### 3.2.3. β-Diversity

**Figure 5:**
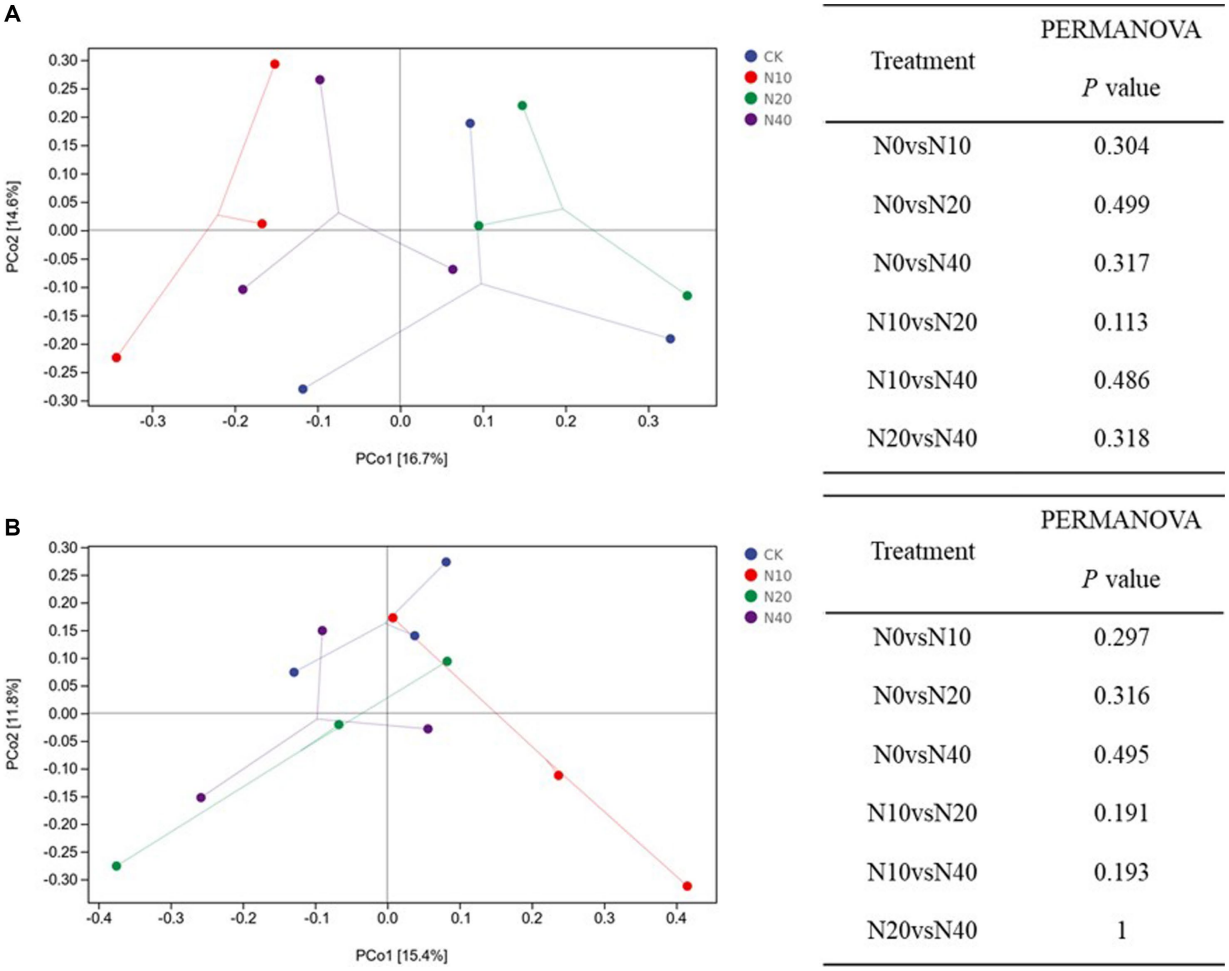
PCoA analysis based on Bray-Curtis distances of **(A)** fungal and **(B)** bacterial communities.

The sum of PCo1 and PCo2 explained 31.3 and 27.2% of the variation in the soil fungal and bacterial communities, respectively (Figure 5). All samples were mainly concentrated in four different areas that corresponded to the four different treatments; this result demonstrates that the bacterial and fungal community structures after the different treatments were heterogeneous and had obvious regional characteristics. The distance matrix of fungal is relatively scattered, and most of the distances between the four groups were similar. Under different N treatments, the similarities in the composition, structure, functional characteristics, and other aspects between the two communities was very high, and the range of change was small. In the bacterial community, only the samples from the N10 treatment group were heterogeneous and showed distinct regional characteristics.

PERMANOVA showed that the structural compositions of the fungal and bacterial communities did not differ significantly among the different treatments (*p* > 0.05).

#### 3.2.4. Effects of N addition on abundance of functional genes for soil N transformation

**Figure 6:**
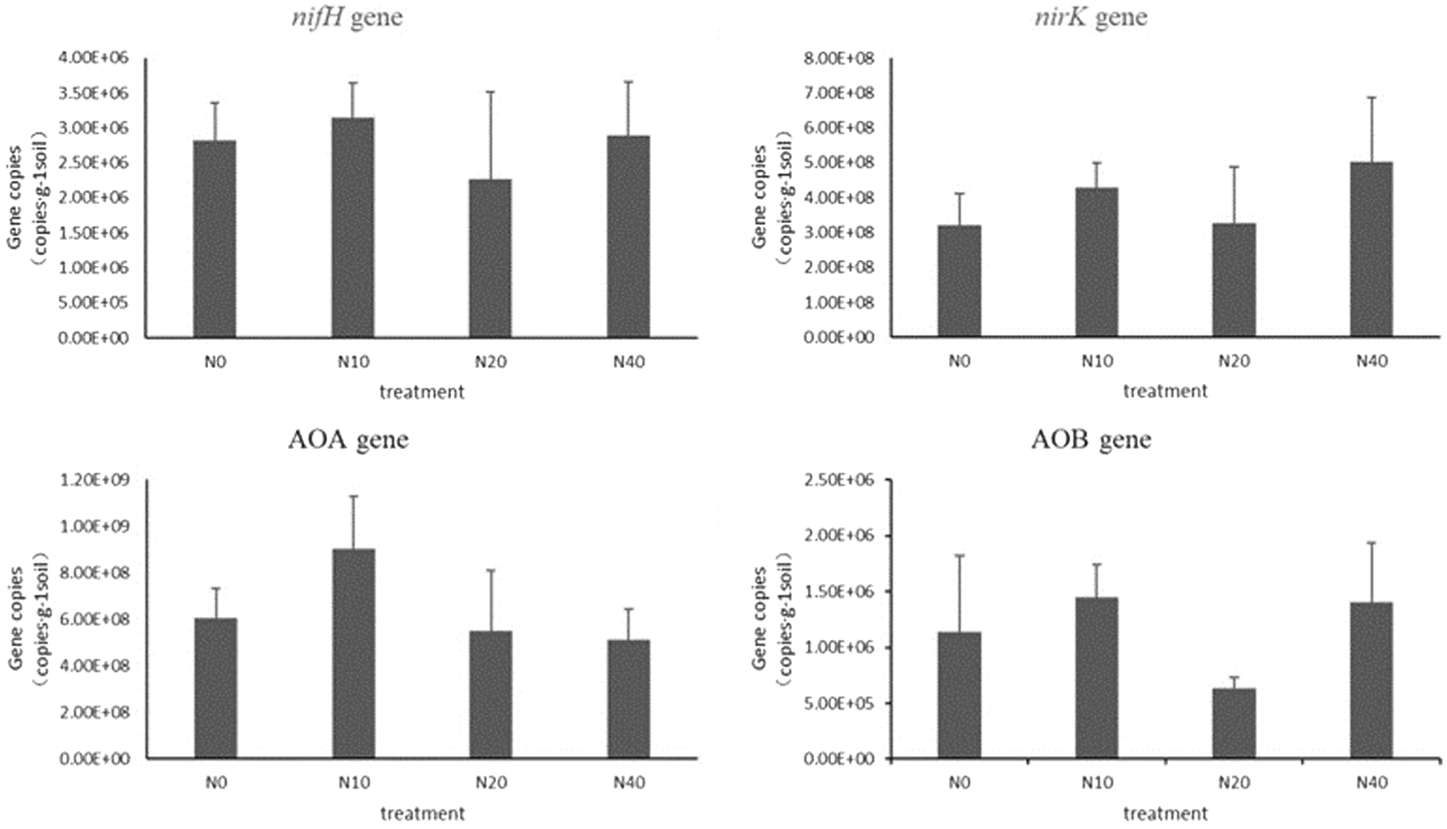
Abundances of functional genes for soil nitrogen N transformation.

Figure 6 shows the abundance of genes related to soil N transformations. The abundance of the *nifH* gene varied in the range of 2.27 × 10^6^–3.15 × 10^6^ copies·g ^−1^ soil. With the initial increase in the N application level, the abundance of *nifH* increased, followed by a decrease after the N20 treatment, and an increase after the N40 treatment. The abundance of *nifH* was the highest under the N10 treatment. The abundance of *nifH* at N10 and N40 was higher than that in the control treatment (N0). The abundance at N20 was lower than that of N0; however, no significant differences were noted among the treatments.

The abundance of the *nirK* gene varied in the range of 3.28 × 10^8^–5.23 × 10^8^ copies·g ^−1^ soil. The abundance of *nirK* increased at N10, decreased at N20, and increased at N40. The highest abundance was observed at N40. Overall, the abundance of *nirK* at different treatments was higher than that at N0, but the differences were not significant.

The abundance of the AOA gene varied in the range of 5.12 × 10^8^–9.03 × 10^8^ copies·g ^−1^ soil. The AOA gene abundance in the N10 treatment was higher than that in the control, while that in other N treatments was lower than that in the control. The results indicated that the AOA gene abundance decreased with an increase in the N supplementation level. The abundance of the AOB gene varied in the range of 6.37 × 10^5^–1.44 × 10^6^ copies·g ^−1^ soil. Compared to the control, the N10 and N40 treatments resulted in higher AOB gene abundance, but the differences were not significant. The copy numbers of the AOB gene in the soil were much lower than those of the AOA gene.

#### 3.2.5. Network diagrams of the soil bacterial and fungal communities

**Figure 7:**
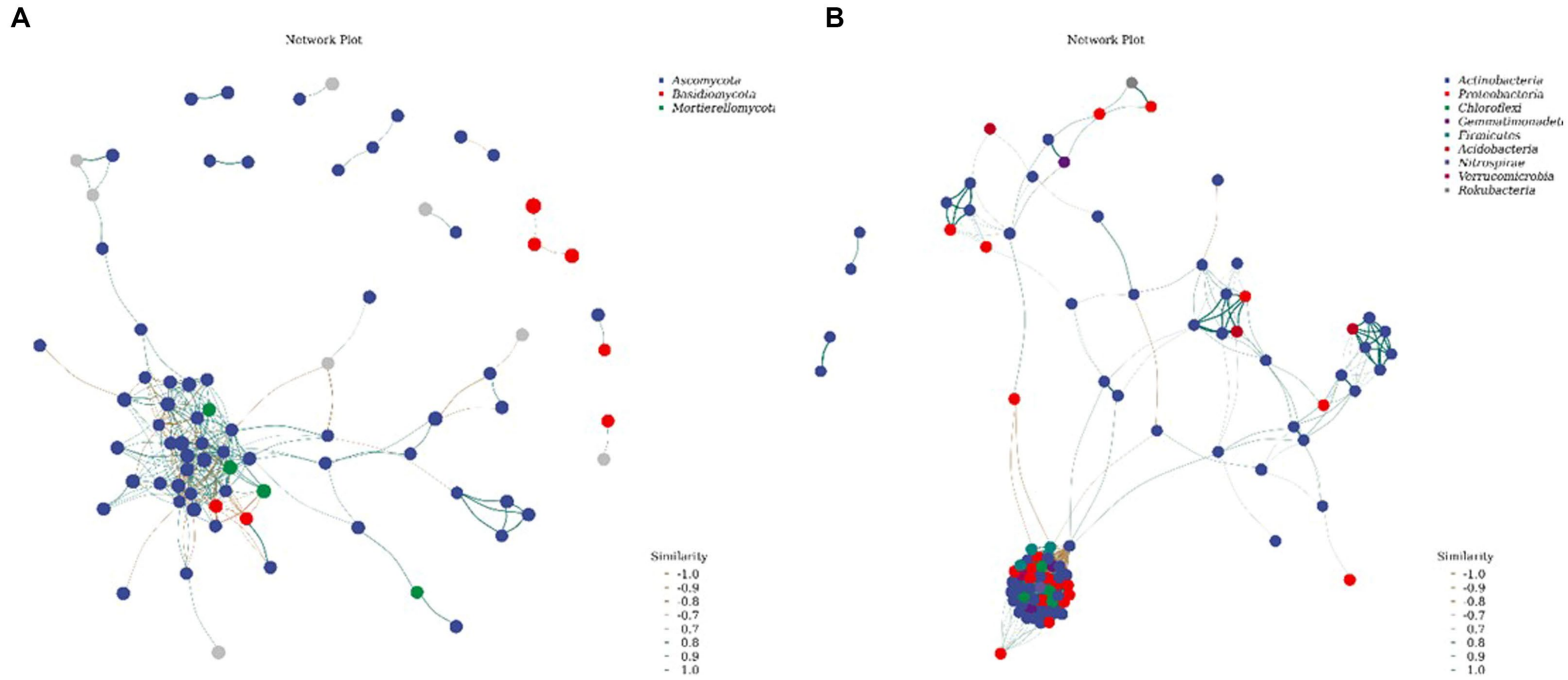
Network of **(A)** fungal and **(B)** bacterial communities.

The species in the network diagram of the soil bacterial communities in the northwest Liaoning grassland were more active and formed more associations than those in the fungal community network (Figure 7). As presented in Table 4, the topological parameters of the network showed that the soil bacteria network contained 1913 nodes and 88,416 edges, while the soil fungi network contained only 307 nodes and 813 edges. Importantly, the nodes in the soil bacteria network were more closely connected than those in the soil fungi network. Therefore, the northwest Liaoning grassland is mainly dominated by soil bacteria.

**Table 4:** Network topology parameters of soil bacterial and fungal communities.

	Average nearest neighbor degree	Average path length	Number of vertice	Number of edge	Modularity
Bacterial communities	99.8906	2.417	1913	88,416	0.5766
Fungal communities	6.8056	5.6201	307	813	0.7234

### 3.3. Correlation among soil variables and microbial community properties

The content of the available phosphorus (AP) in the soil and the Good’s coverage index were significantly positively correlated (*p* < 0.05) (Table 5), indicating that fungal coverage increases with increasing soil AP content. The content of the soil organic carbon (SOC) was significantly negatively correlated with the Chao1 and observed species indices (*p* < 0.05), indicating that the number of fungal species in the soil decrease with an increase in SOC content.

**Table 5:** Correlation analysis between environmental factors and microbial diversity under different nitrogen addition treatments.

		Chao1	Shannon	Simpson	Pielou	Observed	Goods
TN	f	−0.253	0.298	0.298	0.287	−0.236	0.203
(g∙kg^−1^)	b	0.066	0.084	−0.037	−0.058	0.179	−0.105
NO_3_^+^-N	f	−0.229	−0.691*	−0.667*	−0.613*	−0.316	−0.435
(mg∙kg^−1^)	b	−0.213	−0.398	−0.498	−0.381	0.153	−0.031
AN	f	−0.334	0.014	0.014	0.084	−0.341	0.162
(mg∙kg^−1^)	b	0.111	−0.277	−0.217	−0.439	−0.014	−0.22
NH_4_^+^-N	f	−0.167	−0.197	−0.197	−0.12	−0.154	0.168
(mg∙kg^−1^)	b	0.202	0.399	0.366	0.317	0.317	−0.18
TP	f	−0.353	−0.249	−0.249	−0.047	−0.366	0.187
(g∙kg^−1^)	b	−0.329	−0.078	0.087	0.081	−0.205	0.301
AP	f	−0.405	0.127	0.127	0.219	−0.357	0.608*
(mg∙kg^−1^)	b	−0.342	0.188	0.1	0.389	−0.089	0.421
SOC	f	−0.577*	−0.477	−0.477	−0.352	−0.609*	0.125
(g∙kg^−1^)	b	−0.408	−0.325	−0.14	−0.027	−0.474	0.4
pH	f	0.44	0.074	0.074	0.063	0.492	0.092
	b	0.336	0.862**	0 0.929**	0.802**	0.566	−0.226
EC	f	−0.329	0.178	0.178	0.07	−0.328	0.151
(S∙m^−1^)	b	0.575	0.327	0.168	0.018	0.492	−0.548
NMR	f	−0.287	−0.442	−0.278	−0.331	−0.333	−0.08
	b	0.007	−0.127	−0.177	−0.15	−0.023	0.265

*Represents significant. **Represents very significant. “f” Represents fungi; “b” Represents bacteria.

The soil pH was positively correlated with the Shannon, Simpson, and Pielou’s indices (*p* < 0.05). The soil alpha diversity index was not significantly correlated with TN, AN, NH_4_^+^-N, TP, AP, and SOC contents, and EC. These findings suggest that pH affects the structure of the soil bacterial community. The addition of N changes the soil pH, thus changing the structure of the soil bacterial community.

**Figure 8:**
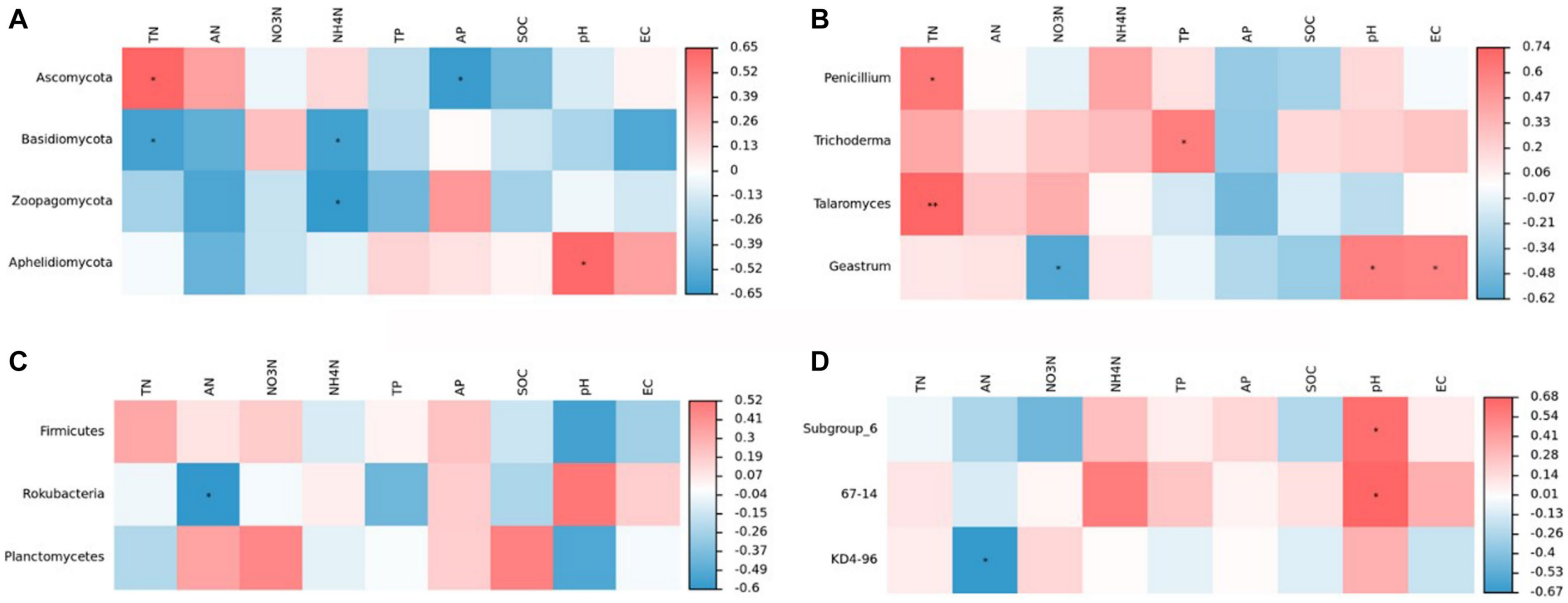
Correlation analysis of community composition of soil fungi **(A,C)** and bacteria **(B,D)** at phyla and genus levels with soil physicochemical properties.

Figure 8 shows the results of the correlation analysis between microbial communities and environmental parameters. The abundance of Ascomycetes was positively correlated with TN, AN, and NH_4_^+^-N contents and significantly positively correlated with TN content (*p* < 0.05). In contrast, the abundance of Ascomycetes was negatively correlated with TP, NO_3_^+^-N, AP, and SOC contents and pH and significantly negatively correlated with AP content (*p <* 0.05). The abundance of Basidiomycetes was negatively correlated with all environmental factors except NO_3_^+^-N content and significantly negatively correlated with TN and AN contents (*p <* 0.05). Zoopagomycota was positively correlated with AP, negatively correlated with other environmental factors, and significantly negatively correlated with AN (*p* < 0.05). Aphelidiomyceta was significantly positively correlated with the soil pH, positively correlated with TP, AP, and SOC contents and EC, and negatively correlated with other environmental factors.

The genus *Penicillium* was most significantly affected by TN content (*p* < 0.05) and negatively correlated with AP and SOC contents. The abundance of *Trichoderma* was significantly affected by TP content (*p* < 0.05) but negatively correlated with AP content. In the case of the genus *Geastrumz*, we observed significantly positively correlation with the soil pH and EC and significantly negatively correlation with NO_3_^+^-N content. In this experiment, changes in environmental factors affected the diversity and structural composition of the soil fungal community.

**Figure 9:**
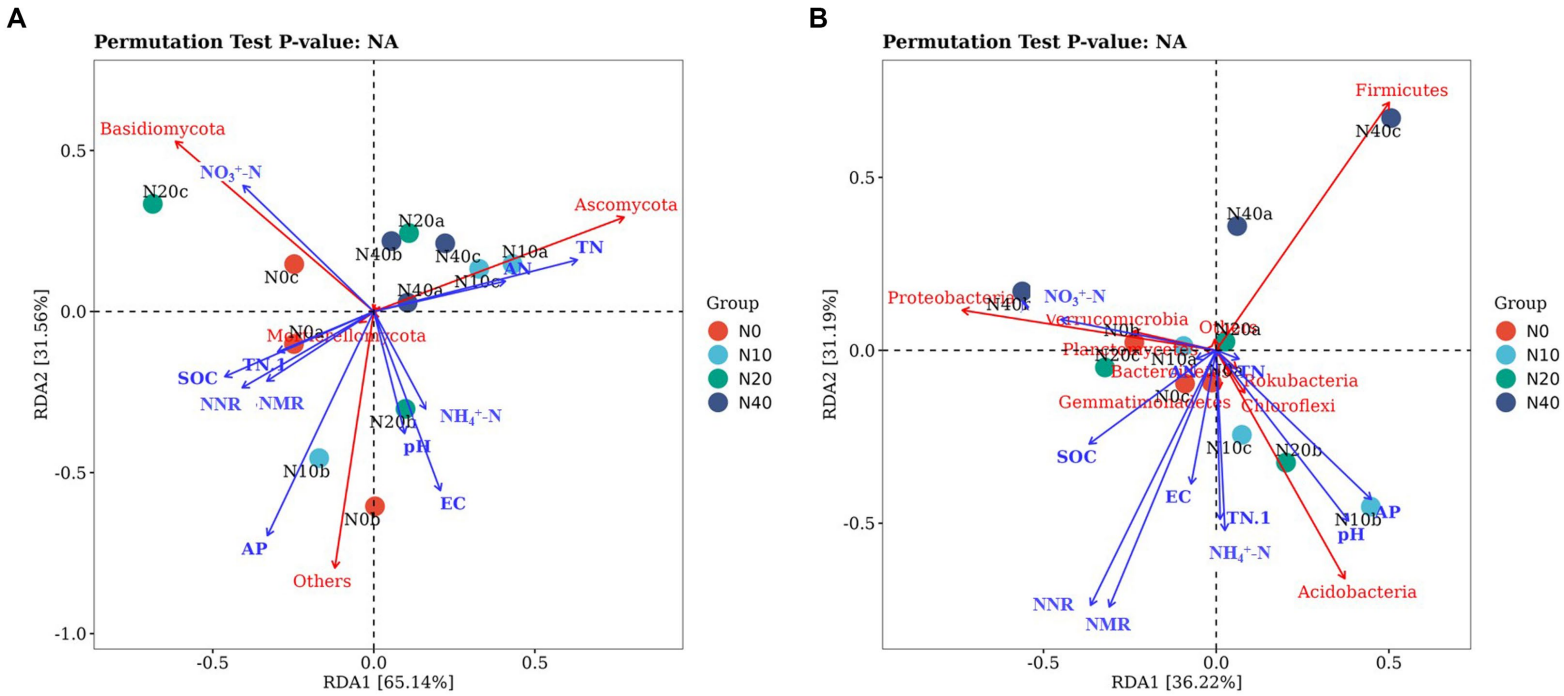
Redundancy analysis (RDA) of the relationship between fungal **(A)** and bacterial **(B)** communities and environmental factors.

We used RDA to examine the effects of soil properties on the microbial communities. The first and second axes in the RDA of the fungal communities explained 61.43 and 31.45% of total variation, respectively (Figure 9). Basidiomycota were positively correlated with soil SOC, NO_3_^+^-N, and TP, and negatively correlated with soil AP, AN, TN, NH_4_^+^-N, soil pH, and EC. Ascomycota had a positive correlation with soil AN and TN contents and NH_4_^+^-N, no correlation with soil EC, and a negative correlation with soil AP, pH, TP, SOC, and NO_3_^+^-N.

The first and second axis in the RDA analyses of the bacterial communities explained 33.71 and 19.47% of the total variation, respectively. The different N treatments were distributed in all quadrants in the figure, while the CK treatment was mainly concentrated in quadrant IV. RDA analysis of the bacteria showed that soil AN, EC, and soil pH were positively correlated with Actinobacteria. Actinobacteria had a negative correlation with soil SOC, NH_4_^+^-N, NO_3_^+^-N, TN, and AP contents. Proteobacteria had a positive correlation with soil SOC and NO_3_^+^-N, no correlation with TN, and a negative correlation with soil AN, AP, TP, NH_4_^+^-N, pH, and EC. Acidobacteria was positively correlated with soil TP, AP, TN, pH, NH_4_^+^-N, and EC, and negatively correlated with soil SOC, AN, and NO_3_^+^-N. The NO_3_^+^-N and TN were significantly correlated with species composition of soil fungal community, The pH and AP were significantly correlated with species composition of soil bacteria community.

The correlation between the gene abundance of soil N-transforming functional microorganisms and soil physical and chemical properties is presented in Table 6. The gene abundance of N-fixing functional genes, denitrification functional genes, and AOB of ammonia-oxidizing bacteria were not affected by environmental factors, while the AOA gene abundance of ammonia-oxidizing archaea showed a significant positive correlation with ammonium N.

**Table 6:** Correlation analysis between functional genes and physicochemical properties.

	*nifH*	*nirK*	AOA	AOB
TN(g∙kg^−1^)	0.784	0.662	0.786	0.761
NO_3_^+^-N(mg∙kg^−1^)	−0.431	0.426	−0.31	−0.26
AN(mg∙kg^−1^)	−0.087	0.292	0.409	−0.08
NH_4_^+^-N(mg∙kg^−1^)	0.437	−0.074	0.970*	0.239
TP(g∙kg^−1^)	0.053	−0.077	0.752	−0.087
AP(mg∙kg^−1^)	−0.085	−0.65	0.669	−0.334
SOC(g∙kg^−1^)	0.347	−0.007	0.925	0.177
pH	0.26	−0.617	0.688	−0.005
EC(S∙m^−1^)	0.635	−0.181	0.992	0.408

*Represents significant.

## 4. Discussion

### 4.1. Effects of N addition on soil properties

In this study, the addition of N had several effects on soil physical and chemical factors. A decreasing trend on the soil pH values was observed with the increase in the supplemented N levels; this effect may be due to the change in ion concentrations caused by N addition, resulting in decreasing pH values at high N concentrations. This is consistent with the results by Jieqiong et al. (2014) on the application of N in a desertification steppe. Compared with the control group, TN content increased significantly only at a low N concentration (N10) because the added N likely has multiple effects on the growth, composition, and function of microorganisms. Excess N inhibits the growth of organisms; therefore, it may be the absorption and utilization by microorganisms and plants that maintains the soil TN content relatively stable. Different N supplementation levels had different effects on the soil chemical properties. A significant increase in TN, TP, and SOC contents was noted under the N10 treatment, while no significant differences in SOC and nitrate N contents between CK and other N concentrations were identified. In contrast, Liu Xing et al. showed that SOC content increased with an increase in the level of applied N because the increase in N input improved the above-ground productivity of grassland plants as well as the amount of plant litter and root exudates, thus promoting the accumulation of SOC. The discrepancy with our results may be because the effect of short-term N application is not immediately obvious and requires further investigation.

### 4.2. Effects of nitrogen addition on microbial diversity and community structure

Ascomycetes and actinomycetes were the common dominant phyla in the fungal communities under different N treatments (Figure 2). This is similar to the composition of the fungal communities in the Hulunbuir sand area, Loess Plateau grassland, and Inner Mongolia desert grassland, but substantially different from their bacterial community structure. This discrepancy may be due to the grassland restoration process in northwest Liaoning. At the same time, the higher the number of connections in the soil microbial community network, the higher its stability and the stronger its ability to inhibit pathogen invasion. In this study, the degree of connectivity of the soil bacterial network was much higher than that of the fungal network (Figure 6), indicating that the bacterial community exhibited strong resistance to invasion. This result suggests that the bacterial community is dominant in the forest steppe ecotone in northwest Liaoning. Bacterial and fungal communities exhibit different sensitivities to N addition (Zhou et al., 2020). Overall, the addition of N had no significant effect on N-transforming microorganisms.

The correlation analysis between environmental factors and N-transforming microorganisms showed that NH_4_^+^-N had a significant effect on AOA gene copies. The addition of nitrogen resulted in the change of soil properties in many aspects. With the increase of added nitrogen concentration, NH_4_^+^-N content in soil showed a gradual increasing trend, and soil pH value decreased significantly. AOA and AOB showed a trend of first increase and then decrease, and showed the maximum value under N10 treatment. It was common for nitrogen accumulation to lead to their quantity increase. Li also confirmed that both the changes of ammonium and NH_4_^+^-N would change the community compositions AOB and AOA (Li and Gu, 2013). In this study, the decrease of AOA and AOB quantity indicated that high concentration of ammonia had an inhibitory effect on ammonia oxidation activity. Whether the decrease of AOA amount caused by the addition of high nitrogen concentration is related to the decrease of ammonia oxidation activity remains to be studied. In our study, the diversity and community structure of microorganisms with nitrogen transformation function were not measured, but only the gene copy number was measured, and the specific changing strains could not be known. Some study showed that nitrogen addition had an impact on diversity and community structure (Wang et al., 2015), so future experiments will focus on it.

### 4.3. Changes in soil TN content and soil pH alter the soil microbial community

In the natural environment, the available N may be limited (the diffusion ability of N in soil is hindered), while the addition of exogenous N may alleviate this restriction (Plett et al., 2020). Many studies have been conducted worldwide on the impact of N addition on soil microbial diversity. The general conclusion was that N addition reduced soil microbial diversity in grassland ecosystems (Wang C. et al., 2018). N addition would lead to competition among soil microbial species; thus, those species with high rates of N utilization that rapidly absorb N and grow after N addition inhibit other species (Bobbink and Willems, 1987), resulting in the loss of some soil microbial species, and thus reduced soil microbial diversity (Sui et al., 2015). Overall, the richness and diversity of soil bacteria first decreased and then increased with an increase in N application rate. The richness and diversity of soil bacteria decreased at medium N application rates and increased at high N application rates; however, the richness and diversity of soil bacteria were lower than those without added N (Li et al., 2020). These results suggest that N addition can reduce the soil microbial diversity in grassland ecosystems. However, some studies have shown that, at different gradients of N addition, the diversity of soil microorganisms increases at low and decreases at high N concentrations. The different effects of N addition on soil microbial communities may be due to the complex response of soil microorganisms to N addition. On the one hand, the addition of an appropriate amount of N alleviates the N limitation in grassland ecosystems and promotes the growth of the above-ground vegetation and the quantity and quality of litter, thus increasing the input of organic matter into the soil. Therefore, abundant resources enable the coexistence of a large number of species. On the other hand, species with a high N utilization rate will rapidly absorb N and grow after N addition, inhibiting the growth of other species; thus, in a high-N environment, loss of some soil microbial species and reduced soil microbial diversity are expected.

The correlation analysis between soil microbial functional diversity and environmental factors showed that the soil fungal Shannon index was significantly negatively correlated with soil NO_3_^+^-N, indicating that the soil N supply affected fungal functional diversity. This effect may be due to the increased NO_3_^+^-N content and free hydrogen ions in the soil after N addition, leading to soil acidification. It is generally believed that soil bacterial communities tend to be more uniform at neutral pH conditions, with higher degrees of richness and diversity. The significant positive correlation between the soil bacterial Shannon index and pH values indicates that continuous N addition may cause a decrease in pH values, leading to an overall decrease in the soil microbial functional diversity. Soil microbial growth was affected by soil environmental factors, among which the degree of soil acidity had the most significant effect (Wang et al., 2021). Correlation analysis between the dominant flora and soil chemical factors showed that soil pH values were significantly positively correlated with the relative abundance of Aphelidiomycota but significantly negatively correlated with the relative abundance of Aphelidiomycota. Additionally, soil pH values were positively correlated with the relative abundance of *hexae*, while negatively correlated with the relative abundance of Firmicutes and *Aspergillus*. The results showed that the soil pH was the main factor affecting the soil bacterial community, consistent with previous studies (He et al., 2017). In general, soil microbial diversity is the highest when soil pH is near neutral, and the presence of excess acid or alkali will put the growth of the microbial community under stress, thus reducing microbial diversity. Previous studies have suggested that the ecological selection of microbial communities by soil acidification and the adaptive evolution of different microbial communities under pressure at acidic conditions are key mechanisms that lead to changes in soil microbial communities (Yan et al., 2017). Soil bacteria are more sensitive to the soil environment than fungi.

## 5. Conclusion

In conclusion, N addition had significant effects on soil TN content, ammonium N content, and pH.N addition had no significant effect on the diversity of fungi and N-transformed microorganisms at the sample site but had a significant effect on the bacterial diversity. TN and pH were the main causes of changes in the soil microbial community. Our study shows that microbial bacteria dominate the forest steppe ecotone in northwest Liaoning Province, and bacteria can better reflect microbial changes under nitrogen application than fungi. Under the background of relatively limited overall understanding of soil microorganisms in the forest steppe ecotone of northwest Liaoning Province, this study explored the effects of nitrogen addition on soil microbial community structure and diversity and its ecological adaptation mechanism, which not only theoretically enriched the research content of forest steppe ecotone. It is also of great significance for the comprehensive assessment and prediction of the response of forest grassland interlacing to global change, the protection of biodiversity and the maintenance of ecosystem balance. The diversity and community structure of microorganisms related to nitrogen conversion function in the experiment may further help us understand the ecosystem, and future research will focus on this.

## Data availability statement

The DNA sequences in this study have been deposited in the National Center for Biotechnology Information (NCBI) Sequence Read Archive (SRA) database under accession number PRJNA949941.

## Author contributions

BR, LB, and JL designed this study. GB, CT, and FY assisted in the field experiment. DL, XM, JY, and MM performed the laboratory analysis. DL and BR wrote the manuscript. All authors reviewed the manuscript and approved for publication.

## Funding

This study was supported by National Natural Science Foundation of China (32001127).

## Conflict of interest

The authors declare that the research was conducted in the absence of any commercial or financial relationships that could be construed as a potential conflict of interest.

## Publisher’s note

All claims expressed in this article are solely those of the authors and do not necessarily represent those of their affiliated organizations, or those of the publisher, the editors and the reviewers. Any product that may be evaluated in this article, or claim that may be made by its manufacturer, is not guaranteed or endorsed by the publisher.

## Figures and Tables

**Table:** 

AN	available nitrogen
AOA	ammonia-oxidizing archaea
AOB	ammonia-oxidizing bacteria
AP	available phosphorus
ASVs	amplicon sequence variants
CK	control group
EC	soil electric conductivity
LSD	least significant difference
N	nitrogen
OTUs	operational taxonomic units
qPCR	quantitative PCR
RDA	redundancy analysis
SD	standard deviation
SOC	soil organic carbon
TN	total nitrogen
TP	total phosphorus

## Footnotes

^1^ https://docs.qiime2.org/2019.4/tutorials/
